# Neurologists’ diagnostic accuracy of depression and cognitive problems in patients with parkinsonism

**DOI:** 10.1186/1471-2377-12-37

**Published:** 2012-06-15

**Authors:** Angela EP Bouwmans, Wim EJ Weber

**Affiliations:** 1 Department of Neurology, Maastricht University Medical Centre, PO Box 5800, Maastricht, AZ, 6202, Netherlands; 2 Division of Movement Disorders, Department of Neurology, Maastricht University Medical Centre, Maastricht, Netherlands

**Keywords:** Mood disorder, Dementia, Non-motor, Parkinson’s disease

## Abstract

**Background:**

Depression and cognitive impairment (CI) are important non-motor symptoms in Parkinson’s Disease (PD) and related syndromes, but it is not clear how well they are recognised in daily practice. We have studied the diagnostic performance of experienced neurologists on the topics depression and cognitive impairment during a routine encounter with a patient with recent-onset parkinsonian symptoms.

**Methods:**

Two experienced neurologists took the history and examined 104 patients with a recent-onset parkinsonian disorder, and assessed the presence of depression and cognitive impairment. On the same day, all patients underwent a Hamilton Depression Rating Scale test, and a Scales for Outcomes in Parkinson’s Disease-Cognition-test (SCOPA-COG).

**Results:**

The sensitivity of the neurologists for the topic depression was poor: 33.3%. However, the specificity varied from 90.8 to 94.7%. The patients’ sensitivity was higher, although the specificity was lower. On the topic CI, the sensitivity of the neurologists was again low, in a range from 30.4 up to 34.8%: however the specificity was high, with 92.9%. The patients’ sensitivity and specificity were both lower, compared to the number of the neurologists.

**Conclusions:**

Neurologists’ intuition and clinical judgment alone are not accurate for detection of depression or cognitive impairment in patients with recent-onset parkinsonian symptoms because of low sensitivity despite of high specificity.

**Trial registration:**

(ITRSCC)NCT0036819.

## Background

Depression and cognitive impairment (CI) are increasingly appreciated as important non-motor symptoms in patients with Parkinson’s disease (PD) and related syndromes [1-11]. Early recognition and diagnosis of both is important as treatment may increase quality of life of patients [12-22], but this is reported to be hampered at different levels. Many depressed patients are not aware of their problems [23] and, although caregivers sometimes have better judgment [24], they can also be misled by personality issues [25]. Non-psychiatrist physicians appear not to do much better, as numerous studies have shown that they perform significantly worse than validated questionnaires [26-39]. The presence of CI also leads physicians to overestimate the presence of depression [40].

Non-motor symptoms in PD tend to be under-diagnosed compared to motor problems, which seems logical as the latter are the primary expertise of neurologists. Some studies have compared different instruments to diagnose CI and depression in PD [41-43], but few have studied how accurate the diagnostic process is in normal daily practice [44,45]. Most often, neurologists focus on motor symptoms and evaluate possible CI and depression implicitly during their consultation [46]. These consultations are often too short to conduct a formal validated test for depression and/or CI. We studied this implicit diagnostic process to assess its accuracy in a consecutive series of patients referred for analysis of a parkinsonian disorder of very recent onset. We focused on this patient group as research on this question has hitherto been done only in patients with a well-established diagnosis of PD.

## Methods

The present study was nested in a larger, prospective study testing the diagnostic accuracy of transcranial duplex scanning (TCD) of the substantia nigra (SN) in the brainstem as an instrument to diagnose PD in patients with a parkinsonism of unclear origin [47].

**Figure 1:**
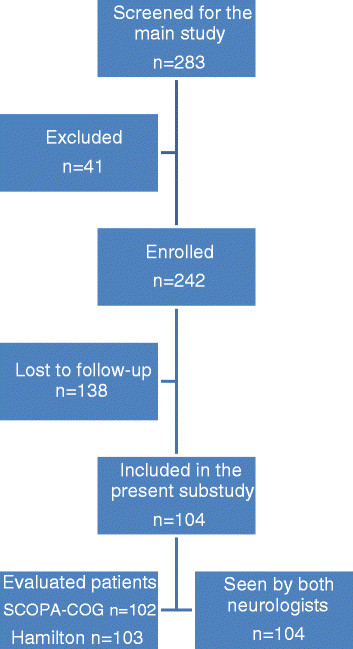
Flowchart of our study.

We invited 283 consecutive patients, who were referred to two neurology outpatient clinics, for analysis of clinically unclear parkinsonian disorder (Neurology Outpatient Clinics of the Maastricht University Medical Centre in Maastricht and of the Maasland Hospital in Sittard, both in The Netherlands). Patients, whose parkinsonism was clearly diagnosable at the first visit, were excluded from the study. For further details see our protocol described elsewhere [47]. Finally, we enrolled 242 patients in our study (see Figure 1) after written informed consent for participation by each patient.

After two years, all patients were re-examined by a pair of neurologists for a final clinical diagnosis. The neurologists were specialists in movement disorders, with more than ten years’ experience in this field. These investigators were blinded for all prior test results from these patients. In planning these visits, we made sure that neither of the two neurologists had ever seen the patient. They were asked to interview and examine the patient, guided by a standard form (see Additional file 1). Family or spouse were allowed to be present at this consultation, but were asked to refrain from answering any questions. Neurologists were not given any objective scales for depression or cognitive impairment. After this consultation, the patient left the room.

The specialists were then asked to indicate on a form if they thought the patient was depressed and if CI was present. There was no communication between the raters on this decision. They were also asked to reach an independent clinical diagnosis, and record it. After this, they were asked to discuss the patient, and reach a final, consensus diagnosis, which served as the “gold standard” in the TCD study [47]. On the day of this evaluation, all patients were asked to complete the Hamilton Depressing Rating Scale (in the text further shortened to ‘Hamilton’) and the Scales for Outcomes in Parkinson’s Disease-Cognition (SCOPA-COG). Both tests have shown their reliability and validity as instruments to assess either depression or CI, both with and without PD [48-53]. The maximum possible score on the SCOPA-COG is 43. A score of 20 or lower is defined as CI [52,53]. The maximum possible score on the 17 item Hamilton is 52. A score 0 to 7 on the Hamilton implies normal/-borderline mood, score between 8 and 15 indicates a mild depression; a score in the range of 16 to 26 indicates a moderate depression; and a score of 27 or higher implies severe depression [50,51]. The results of these tests were not provided to the patients. We also asked the patients the following questions: ‘Do you think you have more difficulties with your memory than people of the same age as you?’ and ‘Did you experience feelings of depression, most of the time during the last month?’ to evaluate their insight about the presence of CI and depression.

The primary objective of this study was to define the accuracy, namely, the sensitivity and specificity of the neurologists’ clinical judgement regarding the presence of CI and depression. The secondary objective was to evaluate the degree of agreement on the diagnosis of CI and depression between the patient himself and the two neurologists. SPSS 16.0 for windows (SPSS, Chicago, IL, USA) was used for statistical analysis. The accuracy of the clinical judgement of the neurologists was evaluated by the means of specificity and sensitivity. The inter-rater agreement was evaluated by the Kappa statistics.

## Results

We had originally enrolled 242 patients into the TCD study (see Figure 1). Follow-up after two years took place between September 2008 and September 2010. Thirty patients (12,4%) had died and 108 patients (44,2%) were unable or unwilling to undergo a second evaluation. The group lost to follow up were significantly older (p = 0,034) and had a higher mean Unified Parkinson’s Disease Rating Scale (UPDRS) at enrollment (p = 0,031). However there were no significant differences in the distribution of the final diagnoses compared to the patients included in the present study. No correlation was found between either the UPDRS scores or results on the Hamilton and SCOPA-COG and the final diagnoses after two years follow-up.

For the present study, 104 patients (65 male, 39 female) were evaluated. The mean age was 70,3 (range 44–90) years. After two years follow-up, 62,5% of the patients used antiparkinson medication,13,5% antidepressants, and 3,8% neuroleptics. No one used cognitive enhancers. The final clinical diagnosis was PD in 53 (51%) patients. For further patient characteristics, see Table 1. The diagnostic groups were demographically similar. The remaining 15 (14%) patients without parkinsonism had alternative diagnoses, such as, isolated tremor, orthostatic tremor, tardive dyskinesia, multi-infarct dementia, Alzheimer disease, stroke, hypoxic encephalopathy, and psychogenic disorder.

**Table 1:** Patient characteristics (data as mean and SD) or count (%)

	**All patients(n = 104)**	**PD** ** (n = 53)**	**APS** ** (n = 13)**	**VP** ** (n = 8)**	**ET** ** (n = 11)**	**DIP** ** (n = 4)**	**No parkinsonism** **(n = 15)**
Mean age in years (SD)	70,3 (9,46)	69,81 (9,74)	70,29 (7,42)	73,71 (1,60)	67,91 (10,38)	67,50 (12,61)	72,44 (11,06)
Men	64%	63%	75%	56%	91%	50%	47%
Mean score UPDRS-III (SD)	15,03 (8,08)	16,88 (6,06)	19,57 (9,94)	18,29 (9,55)	6,82 (3,95)	17,00 (10,86)	8,75 (6,18)
SCOPA-COG >20 (no CI)	55%	58%	67%	38%	82%	25%	60%
SCOPA-COG < =20 (yes CI)	45%	42%	33%	62%	18%	75%	40%
Hamilton < 8 (no depression)	74%	81%	54%	71%	73%	100%	60%
Hamilton > =8 (yes depression)	26%	19%	46%	29%	27%	0%	40%
‘Self-diagnosed’ presence of CI	38%	34%	69%	25%	18%	50%	47%
‘Self-diagnosed’ presence of depression	18%	13%	23%	13%	36%	25%	20%

PD = Parkinson’s disease, APS = atypical parkinsonian syndromes, VP = vascular parkinsonism, ET = essential tremor, DIP = drug induced parkinsonism, UPDRS-III = Unified Parkinson’s Disease Rating Scale part III, SCOPA-COG = Scales for Outcomes in Parkinson’s Disease-Cognition, Hamilton = Hamilton Depressing Rating Scale CI = cognitive impairment.

### Cognitive impairment

In total 102 patients were able to complete the SCOPA-COG, two patients were too tired after the consultation with the neurologists, and therefore, were not able to complete this test. The mean SCOPA-COG score was 21,1 with a range of 4 to 36. See Table 2 for the results on CI ‘diagnoses’ by the neurologist compared with the SCOPA-COG scores.

**Table 2:** Results of neurologists’clinical assessment and patients’ insight versus SCOPA-COG and Hamilton scores (data in numbers)

	**Rater 1**	**Rater 1**	**Rater 2**	**Rater 2**	**Patient**	**Patient**	**Patient**
	**yes**	**no**	**yes**	**no**	**yes**	**no**	**do not know**
SCOPA-COG > 20 (no CI)	4	52	4	52	7	45	4
SCOPA-COG < =20 (yes CI)	16	30	14	32	12	32	2
Hamilton < 8 (no depression)	7	69	4	72	21	49	6
Hamilton > =8 (yes depression)	9	18	9	18	19	7	1

SCOPA-COG = Scales for Outcomes in Parkinson’s Disease-Cognition, Hamilton = Hamilton Depressing Rating Scale, CI = cognitive impairment.

The sensitivity of neurologist 1 for ‘diagnosing’ CI was 34,8% and of neurologist 2 30,4% (see Table 3). Specificity of both neurologists was 92,9%.

**Table 3:** Summary of the neurologists’ and patients’ diagnostic performance

	**CI**	**Depression**
Rater 1 specificity	92.9%	90.8%
Rater 1 sensitivity	34.8%	33.3%
Rater 2 specificity	92.9%	94.7%
Rater 2 sensitivity	30.4%	33.3%
Agreement between two neurologists kappa (95% confidence interval)	0.74 (0.57–0.91)	0.62 (0.39–0.85)
Patient specificity	86.5%	70.0%
Patient sensitivity	27.3%	73.1%
Neurologist 1-patient agreement kappa (95% confidence interval)	0.34 (0.12–0.56)	0.43 (0.27–0.60)
Neurologist 2-patient agreement kappa (95% confidence interval)	0.44 (0.23–0.66)	0.28 (0.12–0.43)

In answer to the question ‘Do you think you have more difficulties with your memory than people of the same age as you?’, 19 (18,6%) said ‘yes’, 77 (75,5%) said ‘no’, and 6 (5,9%) ‘I do not know’. See Table 2 for the results on this ‘self-diagnosis’ of the patients on CI combined with their SCOPA-COG scores. Because six patients could not answer the question with ‘yes’ or ‘no’, we excluded them from this analysis. Therefore the sensitivity of self-diagnosis of CI by the patient was 27,3%. Its specificity was 86,5%.

The agreement between neurologist 1 and neurologist 2 was good with a kappa value of 0,74 (95% confidence interval 0,57–0,91) (see Table 3). However, the agreement between the neurologists and the patient was much lower with a kappa value varying between 0,34 (95% confidence interval 0,12–0,56) and 0,44 (95% confidence interval 0,23–0,66).

### Depression

In total, 103 patients were able to complete the Hamilton (one patient was too tired after the consultation and was not able to complete this test). The mean Hamilton score was 5,5 with a range of 0 to 26. In this study, none of the patients had a score of 27 or higher. We defined depression at the total score of 8 or higher on the Hamilton.

See Table 2 for the results on the depression diagnosed by the neurologists compared to the patients’ Hamilton score. The sensitivity of depression diagnosis by both neurologists was 33,3%, specificity of neurologist 1 was 90,8% and of neurologist 2 was 94,7% (see Table 3).

In answer to the question ‘Did you experience feelings of depression, most of the time this last month?’, 40 (38,8%) said ‘yes’, 56 (54,4%) said ‘no’, and 7 (6,8%) ‘I do not know’. See Table 2 for the results on ‘self-diagnosis’ by the patients compared with their Hamilton score. Because 7 patients could not answer the question with ‘yes’ or ‘no’, we excluded them from this analysis. Therefore, the sensitivity of the patients’ self-diagnosis of depression was 73,1%. Its specificity was 70,0%.

The agreement between neurologist 1 and neurologist 2 was good with a kappa value of 0,62 (95% confidence interval 0,39–0,85) (see Table 3). Agreement between neurologists and patients was lower, with a kappa value varying between 0,28 (95% confidence interval 0,12–0,43) and 0,43 (95% confidence interval 0,27–0,60).

## Discussion

We studied the accuracy of neurologists’ ability to diagnose depression and CI in patients with parkinsonian symptoms, in a way that most closely resembles normal daily clinical neurology practice, as a definite diagnosis of a parkinsonian syndrome is often not possible in the first few years. And, while the neurologists in our study were very experienced in PD and spent on average more time per patient than normal, we speculate that their results might even be somewhat inflated.

A limitation of the study is the use of psychometric scales as a proxy for the diagnoses of CI and depression. While these can not, of course, replace a complete diagnostic work-up by a specialised psychiatrist with a psychometric battery, we do feel that the results show that there is probably a considerable underestimation of the presence of these clinical problems also in patients with a very recent-onset of a parkinsonian disorder. Another limitation of our study is the large number of patients lost to follow-up. This could have biased our population towards one with less morbidity, thus increasing diagnostic difficulty. From the age and UPDRS-scores one can infer that PD patients with more severe disease and thus with possibly more severe depression and CI were underrepresented in this study.

We found that this implicit diagnostic process by neurologists is far less accurate than validated tests. The prevalences of both depression and CI found in our study, are representative for the general population of PD patients [54,55].

We found that neurologists underestimated the number of patients with CI, by up to 70%. They did somewhat better than the patients themselves. Compared to other studies, our neurologists’ diagnostic sensitivity for CI was lower than those of general practitioners (GP’s), although both had a high specificity [34-40]. Our data on the recognition of CI are in line with two earlier studies on PD patients, which both found the majority of symptoms going unrecognised and untreated. However, one of those was a retrospective chart review and the other, prospective, study involved older, well-established PD patients. We did not confirm earlier research that the presence of CI leads to overestimating of depression by doctors [40].

In ‘diagnosing’ depression, our neurologists showed low sensitivity and high specificity. ‘Self-diagnosis’ of depression by the patient had a higher sensitivity compared to the neurologists, although the specificity was lower. The neurologists missed up to 67% of the patients with depression. The patients in our study overestimated the presence of depression. Presence of CI had no influence on the insight of the patients on depression. ‘Diagnostic accuracy’ of depression by our neurologists was comparable to GP’s [26], but self-diagnosis of depression in our patient population had a remarkably higher sensitivity than in an earlier study done by Watson [24]. However, that study only included patients with dementia, and this may explain this difference.

## Conclusions

Intuition and clinical judgment are not enough for a neurologist to recognize depression and/or cognitive problems in patients with recent-onset parkinsonian syndromes, such as, PD and atypical parkinsonian syndromes (APS). It is important to realize this, considering the consequences of untreated depression and CI. Our neurologists had a high specificity diagnosing CI and depression, but at the same time missed more than half of the patients with these problems. Patients themselves are not better at self-diagnosing these non-motor symptoms.

## Competing interest

All authors declare that they have no competing interests.

## Authors’ contribution

Both authors have contributed to all of the following: 1. Research project: A. Conception, B. Organization, C. Execution; 2. Statistical Analysis: A. Design, B. Execution, C. Review and Critique; 3. Manuscript Preparation: A. Writing of the first draft, B. Review and Critique. Full financial disclosure for the previous 12 months: Bouwmans: Stichting Internationaal Parkinson Fonds. Weber: Stichting Internationaal Parkinson Fonds. Both authors read and approved the final manuscript.

## Pre-publication history

The pre-publication history for this paper can be accessed here:

http://www.biomedcentral.com/1471-2377/12/37/prepub

## Supplementary Material

Additional file 1 **Standard clinical scorings form [**[2,33-38]. Click here for file
