# Supplementary material for: Neurologists’ diagnostic accuracy of depression and cognitive problems in patients with parkinsonism
Source: BMC Neurol. 2012 Jun 15;12:37. doi: 10.1186/1471-2377-12-37 (PMC3465198; doi:10.1186/1471-2377-12-37)
Supplement: Additional file 1 — Standard clinical scorings form [[2,33-38]. [file 1471-2377-12-37-S1.doc]

**Additional file: Standard clinical scorings form**

**general:**

Age:

Referrer:

Investigator:

Date of investigation:

**Anamnesis:**

1. Medical history:

2. Medication:

Anti-parkinsonian drugs in present and past:

Effect of anti-parkinsonian drugs (positive, negative, unclear, inadequate dose)? Adequate dose of anti-parkinsonian drugs (at least 1000mg l-dopa daily):

3. Intoxications (alcohol, smoking, drugs):

4. Durations of complaints:

5. Most affected body-side:

**CLINIMETRIC SCALES:**

# uk BRAIN BANK criteria [2]:

# United Parkinson’s Disease Rating Scale (part I, III, IV) [33]:

# hoehn and YAHR score [34]:

# SCOPA cognition scale [36]:

# Hamilton rating scale for depression [35]:

# Sniffin sticks smell test [37] [38]:

#

**Most probableclinical diagnosis:**

1. Idiopathic Parkinson’s disease:

2. Essential tremor

3. Vascular parkinsonism

4. Drug-induced parkinsonism

5. Multiple system atrophy

6. Progressive supranuclear palsy

7. Diffuse Lewy body disease

8. Corticobasal degeneration

9. Other:

**DEGREE OF CONFIDENCE:**

Certain / Not certain
